# Preparing safe doctors through innovative methodologies: Importance and cost effectiveness of emphatic patient safety teaching to undergraduates

**DOI:** 10.12669/pjms.42.4.14969

**Published:** 2026-04

**Authors:** Nadeem Alam Zubairi, Fahad Anwer, Fahad Ussif Gadi, Moayyad Salah Baothman, Mohammed Faleh Alahmadi

**Affiliations:** 1 Nadeem Alam Zubairi Department of Pediatrics, Faculty of Medicine, King Abdulaziz University, Rabigh, Saudi Arabia; 2 Fahad Anwer Department of Family & Community Medicine, Faculty of Medicine, King Abdulaziz University, Rabigh, Saudi Arabia; 3 Fahad Ussif Gadi Department of Pediatrics, Faculty of Medicine, King Abdulaziz University, Rabigh, Saudi Arabia; 4 Moayyad Salah Baothman Department of Internal Medicine, Faculty of Medicine, King Abdulaziz University, Rabigh, Saudi Arabia; 5 Mohammed Faleh Alahmadi Department of Pediatrics, Faculty of Medicine, King Abdulaziz University, Rabigh, Saudi Arabia

**Keywords:** Medical errors, Patient safety, Safety culture in medicine, Undergraduate medical training

## Abstract

**Objectives:**

Patient safety is a major concern for all stakeholders in healthcare. Despite increased awareness, incidents of medical errors remain frequent. It seems imperative to inculcate the necessary concepts and practices related to patient safety at the undergraduate level. Preparing safe doctors can reduce medical errors and the related financial burden. This study investigates the impact of modified strategies for teaching and assessing Patient Safety on medical students.

**Methodology:**

The study involved the innovative changes in teaching and assessment methodologies for the Patient Safety module at King Abdulaziz University, Faculty of Medicine, Rabigh, Saudi Arabia, conducted in March 2025. Seventy seven final year students of year 2025 were included and made to be actively engaged in the journey patients undergo during their illnesses, analyzing safety-related issues from initial reporting to the ER/OPD until discharge. Practical sessions and various Patient Safety Improvement Initiatives were included. Assessment was consolidated with additional modalities, emphasizing the practical aspects of safety culture. To verify the impact, pre- and post-module surveys and skill checks were conducted. Finally, all students were subjected to twenty patient safety-related situations, and their responses were recorded.

**Results:**

Statistically significant improvement in knowledge and attitudes was found in post-module survey. Assessment with practical modalities yielded excellent results. More than 90% of students adopted a correct approach when subjected to safety-related situations, including avoidance and post-error handling.

**Conclusion:**

Bringing innovative changes in teaching patient safety and assessment at the undergraduate level is an efficacious way to prepare safe doctors. We recommend a similar approach for Nursing and Paramedical schools.

## INTRODUCTION

Patient safety (PS) is a major concern for all stakeholders involved in healthcare. Despite better awareness, the incidents related to medical errors and professional negligence, throughout the world, are frequent.^1^ The magnitude of the problem can be judged from the fact that in the USA, medical-related errors are 3^rd^ leading cause of mortality after heart disease and cancer.^2^ In the Kingdom of Saudi Arabia (KSA), the number of reported cases of medical neglect is on the rise.^3^ It also has huge financial implications, in the form of treatment costs, costs related to care for the long-term complications originating from the errors, and huge legal expenses. Different strategies have been employed to minimize these preventable errors.^4^ One of the important strategies is to prepare safe doctors from the very start. Therefore, it is imperative to inculcate the necessary concepts and practices related to patient safety at the undergraduate level.^5^ Strengthening the foundation will decrease the chances of errors and will yield cost-effective long-term benefits. The authors have done the same by reforming the Patient Safety module (PSM) at their faculty.

The objective of this study was to investigate the impact of modified teaching, learning, and assessment strategies implemented during the Patient Safety module.

We believe that if this impact is strong, then we will be producing young doctors who are well familiar with different aspects of patient safety and will be conscious about it. They will know how to avoid errors and will be practicing it. They will also know what actions to take in case of a safety issue. In short, they will be nurturing a safety culture at their workplace.

## METHODOLOGY

This pre- and post-intervention study was conducted during the Patient Safety module for final-year medical students at the Faculty of Medicine in Rabigh, King Abdulaziz University, Kingdom of Saudi Arabia (KSA), in March 2025. Seventy-seven students took part in the study. There were 45 male and 32 female students. Completion of the module and appearance in the examination were mandatory as an inclusion criterion. Several innovative changes in teaching methodologies were introduced while retaining the WHO guidelines and covering all stipulated topics. Instead of relying heavily on traditional lectures, students were engaged in a journey through which patients pass during their illnesses, analyzing safety-related issues from initial reporting to the ER/OPD through discharge and follow-up. Interactive sessions were supplemented with six related practical workshops, such as hand hygiene techniques, applying surgical gowns, and proper documentation of medicines. Students were trained to master these skills instead of leaving them for the internship period. They were also given different patient-safety-related innovative assignments, which were both individual and group-based. An on-board and dedicated faculty from various departments was chosen to deliver the module. In the assessment, instead of mainly using an MCQ-based exam, students were assessed through multiple modalities, with an emphasis on practical aspects of safety culture. All workshops were subjected to a pre-and post-assessment of the related skill. Different individual and group-based tasks were part of the evaluation. To verify the impact, pre- and post-module surveys were conducted. The knowledge domain survey had 25 items covering different areas of patient safety, while the attitude domain survey had seventeen items. Improvement in six essential related skills was gauged through the outcome of post-workshop assessments in comparison to the pre-workshop check. Finally, all seventy-seven students were subjected to twenty patient safety-related situations at the completion of the module, and their responses were recorded. These scenarios were carefully created to cover all aspects of patient safety, including avoidance and post-error handling.

### Ethical Approval:

Approval to conduct the study was obtained from the Ethical Committee at March 10, 2026. Informed consents were obtained from students who participated in this study.

### Statistical Analysis:

Data were analyzed using SPSS 21. Frequencies and percentages were used to represent categorical variables, and significance was considered at a P value of *<*0.001.

## RESULTS

**Fig.1:**
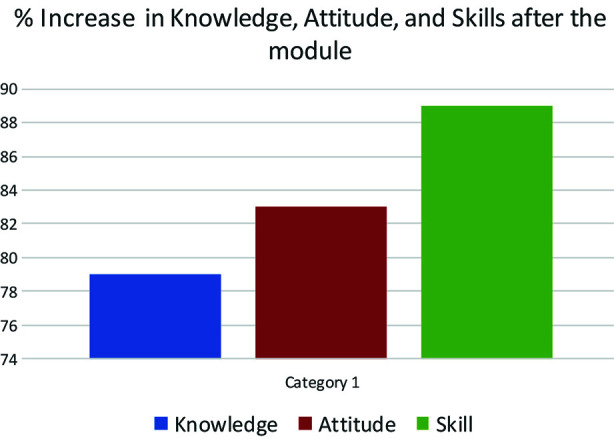
Improvement in Knowledge, Attitude, and Skills on completion of the module.

A statistically significant improvement in knowledge (79%) and attitudes (83%) was recorded in the post-module survey when compared to the pre-module survey. Related six skills were checked before and after the training workshops, and an overall enhancement of 89% was achieved (Fig.1).

**Fig.2:**
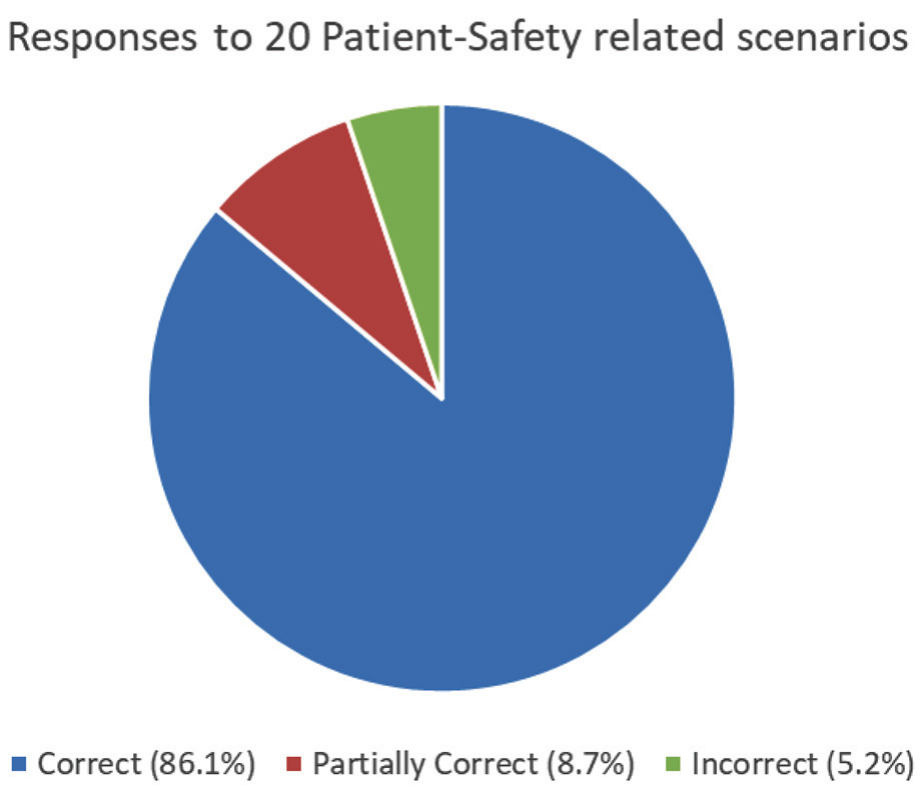
Students’ Responses to 20 Patient-Safety related scenarios.

Breakup of individual skills’ enhancement and cumulative improvement in skills is depicted in Table-I. More than 85% of students adopted a correct approach when subjected to twenty patient-safety-related situations, while around 9% gave partially corrected responses, as shown in Fig.2.

**Table-I:** Pre- and post-module analysis of 77 students’ practical skills regarding Patient Safety.

	Before the start of PSM	At the end of PSM	P-Value
Hand Hygiene	10 (13%)	77 (100%)	<0.001
Surgical Hand Scrub	4 (5%)	75 (97.4%)	<0.001
Applying a sterile gown and donning gloves	2 (2.5%)	74 (96.1%)	<0.001
Documentation of medicines in a patient’s paper	12 (15.5%)	76 (98.7%)	<0.001
Practicing dosage calculation	7 (9%)	77 (100%)	<0.001
Transfusion safety and needle safety	7 (9%)	73 (94.8%)	<0.001
Cumulative improvement	7 (9.09%)	75 (97.8%)	<0.001

## DISCUSSION

The study revealed a significant positive impact on undergraduate medical students of bringing innovative changes in teaching and assessment modalities in the PS module. All areas of learning related to PS, including knowledge, attitude, and skills, were enhanced as evident from the results. The positive impact was further evident from the overwhelmingly correct approaches suggested by the students when exposed to various scenarios related to the safety of the patients. Students who are more exposed, more aware, and better prepared for PS issues are expected to be safer doctors in their practical life.

The need to include patient safety in undergraduate training as a specific domain was gradually recognized over the last two decades, as health givers, health seekers, and authorities responsible for the provision of the healthcare system became aware of the magnitude of the problem. This recognition is evident by a simultaneous increase in PS-related research all over the world.^6^ Limited information was available previously regarding how this important aspect was being covered in medical schools, and until recently, several medical schools did not incorporate an official Patient Safety module into their curricula.^7^

Despite better recognition, there is still no consensus on what to teach, who should teach, and how to teach PS to undergraduate medical students.^8^ Few universities have designed PS teaching based on guidelines provided by the WHO, while others have designed their own methodologies for teaching PS to students.^9^ Curriculum and faculty development for teaching PS is still not standardized and is even insignificant at some places.^10^ Moreover, the effectiveness of these teachings in preparing a safe future doctor remains a matter of debate. One clue is that, despite the realization over the last two decades, the incidence of medical errors is increasing worldwide.^11^ While all necessary steps are required to tackle this situation in hospitals and other healthcare settings by instilling a safety culture, it seems logical to conclude that undergraduate training in this regard is still insufficient and ineffective. This has to be addressed. Additional innovative measures can help in preparing the foundation for safe future doctors.

Our study indicates that a significant improvement in students’ knowledge, attitude, and skills related to patient safety can be brought about through an augmented PSM. Innovative tasks, additional teaching strategies, and improved assessment modalities were employed for this purpose. Improvement in knowledge and attitude related to patient safety has been documented in a few studies,^12^ but these studies primarily employed lecture-based teaching, MCQ-based assessments, and questionnaire-based research. We first augmented the teaching and assessment methodologies, and then, instead of relying only on the questionnaire-based feedback, we included additional tools to see the impact. Practical workshops had a pre-check of existing skills, and at the end, an assessment to ensure the learning and to record the impact. Moreover, after the module, students’ responses to twenty patient safety-related situations were recorded to gauge their understanding, learning, and related decision-making. These enhanced teaching and assessment methodologies and the evaluation of their impact are discussed below.

### Enhanced teaching modalities:

While introducing innovative changes to our teaching and assessment methodologies, we retained the basic framework provided by the World Health Organization (WHO) for the curriculum on teaching patient safety to undergraduate medical students.^13^ Although we adopted WHO guidelines, which remain the backbone in this context,^14^ we supplemented the process by introducing several novel activities to enhance learning and ensure the delivery of Learning Outcomes more effectively. We also brought necessary changes for better delivery of content and optimal learning, as allowed and encouraged by the WHO. It was also modified to cater to local needs and factors prevailing in KSA.^15^ All 11 topics were included, as mentioned in Table-II.

**Table II:** Topics (WHO guidelines for Patient Safety teaching).

Topic 1:	What is patient safety?
Topic 2:	What is a human factor and why is it important to patient safety?
Topic 3:	Understanding systems and the impact of complexity on patient care
Topic 4:	Being an effective team player
Topic 5:	Understanding and learning from errors
Topic 6:	Understanding and managing clinical risk
Topic 7:	Introduction to quality improvement methods
Topic 8:	Engaging with patients and care takers
Topic 9:	Minimizing infection through improved infection control
Topic 10:	Patient safety and invasive procedures
Topic 11:	Improving medication safety

Hospitals all over the world are eager to have a culture of PS but are facing challenges and barriers.^16^ There are many obstacles, like the blame culture and unawareness.^17^ Priming the undergraduate medical students through their full involvement and awareness regarding the patient safety issues in healthcare settings, and what is going on globally and locally in this context, will make the task easier. Future doctors must be apprised and groomed regarding patient safety before actual exposure in the coming years. This is only possible if PS is not only part of the curriculum, but is also learned through innovative teaching methodologies. Below is a brief description of those adopted by the authors and associated faculty, as shown in Table III.

**Table III:** Added teaching modalities in brief.

Practical Workshops:	
Sterilization Workshop	#1 - Hand Hygiene
	#2 - Surgical Hand Scrub
	#3 - Applying a sterile gown and donning gloves
Medication Safety Workshops	#1: Documentation of medicines in a patient’s paper
	#2: Practicing dosage calculation
	#3: Transfusion safety and needle safety
***Practicing patient handovers***	
***Innovations in learning experience***	
*01.Scientific Articles related to patient safety*	[Individual-Based Task]
*02.Case study related to patient safety*	[Individual-Based Task]
*03.Safety issues in one selected medication*	[Individual-Based Task]
*04.Patient Safety Improvement Initiative*	*(PSII) [Group-Based Task]*

### Practical workshops:

Six practical sessions were included to let the students practice the common processes that they will be frequently performing in the future, keeping the aspect of patient safety in mind. Learning after graduation should be continued, but the earlier the better. All workshops had a pre-and post-workshop check, as explained earlier. These practical sessions include:

### Sterilization Workshop:

It covers:

#1 - Hand Hygiene

#2 - Surgical Hand Scrub

#3 - Applying a sterile gown and donning gloves

### Hand wash technique:

Students were explained the importance of washing hands according to the recommended technique, and were given a practical demonstration. It was followed by allowing all students to do it themselves, rectifying their mistakes till the proper procedure is done. This may seem to be very basic, but studies have shown that an alarming percentage of doctors involved in direct patient care are unaware of the proper hand-washing technique or are not practicing it.^18^

Similarly, students were trained for surgical hand scrub, applying a sterile gown, and donning gloves

### Medication Safety Workshops:

***#1: Documentation of medicines in a patient’s paper:*** Medication errors account for more safety issues than any other.^19^ Students were made to practice the documentation of medicines for patients using numerous examples. They were taught how to document it correctly and then checked with examples to ensure the recommended techniques of writing. For example, the use of leading zeros and avoidance of trailing zeros while entering/writing the medicine, clear instructions about the frequency and timings, etc., were practiced
***#2: Practicing dosage calculation:*** Medication errors due to wrong dosage are common even in advanced medical setups.^20^ We led the students to practice different dosage calculations for pediatric preparations according to weight. For some critical drugs, they were asked to calculate according to Body Surface Area. Quick calculations of medicines needed in the ER were also practiced.
***#3: Transfusion safety and needle safety*** were also covered

### Practicing patient handovers:

Proper handover of every patient to the incoming colleague at the end of the duty is an essential and daily process in an intern’s ward life. Errors in handing over the patient properly have led to so many mishaps.^21^ Students practiced that with simulated patients and scenarios. This is part of an effort to enhance the much-needed element of the human factor in PS teaching.^22^

### Additional modalities:

It has been well recognized and documented that teaching PS to undergraduates is not simple and needs different innovative and out-of-the-box activities to ensure effective delivery of related knowledge and skills.^9^ Besides the methodologies of teaching explained above, we incorporated a few innovative activities during the module. These were meant to bring the final year student to full involvement and awareness regarding what is going on globally and locally in the context of Patient Safety. Besides the use of traditional class quizzes and final exams, these innovative modalities of learning were also part of the assessment. These are numerated and explained briefly below:

1. Scientific Articles related to patient safety [Individual-Based Task]
2. Case study related to patient safety [Individual-Based Task]
3. Safety issues in one selected medication [Individual-Based Task]
4. Patient Safety Improvement Initiative (PSII) [Group-Based Task]

1. ***Scientific Articles:*** Each student is required to search for an article on a selected topic related to PS, prepare a summary, and discuss it in detail with the instructor, preferably with reference to a few other articles on the same subject
2. ***Case study:*** Each student has to select a case in the hospital and needs to identify potential safety issues related to the case, with a detailed discussion on one of the possible safety issues and its prevention.
3. ***Safety issues in one selected medication:*** Each student has to select one medication and prepare a report covering indications and dose, cautions, interactions, side-effects, use in pregnancy and lactation, adjustment for hepatic and renal impairment, prescribing and dispensing information, and advice for patient/ caretaker. This is to make them aware of the safety issues related to the use of medicines
4. ***Project related to patient safety:*** We also call this the “Patient Safety Improvement Initiative”. It is a group task. Each group is assigned a patient-safety-related topic, and they prepare and present all necessary issues related to it, including how these can be improved in local settings. Some of the topics include:
  - Infection Control
  - Medication Safety
  - Growing Antibiotic Resistance
  - Safety in Anesthesia and Surgery
  - Safety in the Blood Bank
  - Safety in the Laboratory
  - Safety in Radiology
  - Safety in OPD

Together, these are expected to make the students aware of potential PS threats. For example, there are specific safety issues in outpatient departments.^23^ Similarly, studies have shown that the rejected radiographs needing re-exposure unnecessarily expose the patients to more radiation.^24^ If the students are aware of these or similar issues before graduation, then the chances of errors will be expectedly less in the future.

### Enhanced assessment modalities:

Enhanced teaching modalities were coupled with enhancements in assessment modalities. Instead of being heavily dependent on the end-of-the-module MCQ examination, we included multiple new assessments. Students were not only trained in the practical workshops, but were also required to pass the practical assessments mentioned above. Carefully selected checklists as per standard recommendations were used. Thus, it was ensured that the students can perform personal sterilization, are acquainted with standard ways of prescribing a medicine, and can adjust doses according to the patient’s requirement. Similarly, all assignments, individual and group-based, were part of the final assessment. The famous dictum of assessment driving learning was employed successfully

### Gauging the impact of augmented teaching and assessment methodologies:

To explore the influence of the newly added modalities, we not only used a pre- and post-module survey related to these issues covering knowledge and attitude domains, but also employed two additional activities

- Practical workshops had a pre- and post-workshop check on skills
- Students’ responses to 20 safety-related situations and scenarios after completing the module.

As mentioned in the results, there was a huge improvement in PS-related knowledge (79%) and Attitude (83%), but the most significant increase was in their overall PS-related skills (89%). All six skills related to patient safety showed statistically significant improvement. Together, all of these indicate a markedly successful change in related areas among the students. Further endorsement of this was evident when students were asked to respond to 20 safety-related situations and scenarios

### Responding to 20 safety-related situations and scenarios:

At the end of the module, students were asked to reflect on 20 carefully selected/prepared situations and scenarios involving patient safety. These were covering various safety issues, including errors in ER, documentation, and use of Electronic Health Record, medication errors, infection spread, communication errors with patients and colleagues, to name a few. Since error disclosure has not been up to standard in various studies,^25^ it was also addressed in the scenarios provided. Students were asked to identify the error or potential error, how it could have been avoided, and what to do if it had occurred. Responses were marked as correct, partially correct, or wrong. As mentioned in the results, more than 90% of students adopted a correct approach (Completely correct: 86.1 %, partially correct: 8.7%) when subjected to patient-safety-related situations, including avoidance and post-error handling. Only 5.2% responses were wrong.

### Strength of this study:

The main strength of this study is in providing solutions, in the form of innovative changes in teaching and assessment methodologies, to address the critical issue of less structured patient safety teaching at the undergraduate level in many places.

### Limitations:

The study has the limitation of being done at an undergraduate medical institute with a relatively smaller number of students. Similar studies at colleges with a large number of students can further contribute to highlighting the issue, validating the findings, and exploring the feasibility of similar initiatives.

## CONCLUSION

Despite the recognition of the importance of patient safety in healthcare, the undergraduate teaching in this aspect is still not uniform and deficient in most places. Preparing safe future doctors can reduce the frequency and nature of errors. Innovative methodologies in teaching and assessment are needed to enhance and ensure the successful learning of undergraduate medical students related to Patient Safety issues. The pre- and post-module surveys and checks showed that there was a significant impact on students’ knowledge, attitude, and skills following the introduction of innovative tasks and assessment modalities used in the Patient Safety Module. The overwhelmingly positive impact indicates that medical students will be aware and primed regarding the importance of various aspects related to patient safety before stepping into practical life as doctors, and are expected to be safe doctors and effective team members. This can help decrease the incidence of medical errors in the future, especially if widely adopted. Undergraduates of today should be the ‘Agents of Change’, bringing the much-needed betterment in patient safety concerns by being conscious from the beginning, exercising safe practices, and promoting a culture of safety. We recommend a similar approach for Nursing and Paramedical schools.

### Authors’ Contributions:

**NAZ:** Conceptualization, Manual check of data, writing draft.

**FA:** Data extraction and analysis of data, Manuscript writing.

**FUG:** Data Analysis, Critical revision of Manuscript.

**MSB:** Data extraction and analysis of data, Manuscript writing.

**MFA:** Manual check of data, Critical revision of Manuscript.

All authors have read and approved the final version, they are also responsible and accountable for the accuracy or integrity of the work.
